# Layered double hydroxide of Cd-Al/C for the Mineralization and De-coloration of Dyes in Solar and Visible Light Exposure

**DOI:** 10.1038/srep35107

**Published:** 2016-11-14

**Authors:** Shahid Ali Khan, Sher Bahadar Khan, Abdullah M. Asiri

**Affiliations:** 1 Center of Excellence for Advanced Materials Research, King Abdulaziz University, Jeddah 21589, P. O. Box 80203, Saudi Arabia; 2 Chemistry Department, Faculty of Science, King Abdulaziz University, P. O. Box 80203, Jeddah 21589, Saudi Arabia

## Abstract

Cd-Al/C layered double hydroxide (Cd-Al/C-LDH) and Cd-Sb/C nanocatalyst are reported here for the de-coloration and mineralization of organic dyes. These catalysts were largely characterized by FESEM, EDS, XRD, FTIR, XPS, PL and DRS. The diffuse reflectance data showed a band gap at 2.92 and 2.983 eV for Cd-Al/C-LDH and Cd-Sb/C respectively. The band gap suggested that both catalysts work well in visible range. The photoluminescence spectra indicated a peak at 623 nm for both the catalysts which further support the effectiveness of the respective catalyst in visible range. Both catalysts also showed good recyclability and durability till 4^th^ cycle. Five dyes, acridine orange (AO), malachite green (MG), crystal violet (CV), congo red (CR) and methyl orange (MO) were used in this experiment. Various parameters of different light intensity such as visible, ultraviolet, sunlight and dark condition are observed for the de-coloration of these dyes. The de-coloration phenomenon was proceeded through adsorption assisted phot-degradation. The low cost, abundant nature, good recyclability and better dye removal efficiency make these catalysts suitable candidates for the de-coloration and mineralization of organic dyes.

Dyes industries play an important role in the progress and development of a country and make the human life beautiful. In pre-historical time people used various dyes to make their environment gorgeous. Most of the dyes stuff are categorized on the basis of its coloring properties, solubility, and chemical nature^1^. These dyes are used to color our clothes, food materials and beverages and even make some medicine colored. Literature survey revealed that there are approximately 10,000 commercially available dyes and 7, 00,000 tons are manufactured per annum worldwide^2^,^3^,^4^. During coloring practices most of the dyes were not intact and therefore, a large percentage of these remaining dyes were dumped into the stream. Approximately 10–15% of the dyes stuff are discharge into the environment which are esthetically not favorable^2^. Recently, dyes stuff are of pronounced environmental distress because of their carcinogenicity and mutagenicity^5^. More than 90% of approximately 4000 dyes were experienced in an ETAD survey (Ecological and Toxicological Association of the Dye stuffs) indicating more than 2 × 10^3^ mg/kg LD_50_ values. The highest toxicity were found in diazo and basic dyes^6^. UK make an environmental policies in Sept. 1997 according to which zero synthetic chemical substances are to be released in the marine environment and ensure that the textile industries should treat their effluent before discharging into the water resources^6^. Developed countries and European community become more rigorous to control the dyes stuff from the industrial effluents^7^. Dyes industries contributed to the development of a country, but unfortunately most of the dyes stuff are discharge into our water resources without any treatment^8^. This led to the contamination of underground water resources by passing from soil to water beds and by owing its carcinogenic nature exposing human health and other organisms to high risk. Dyes stuff in minute quantity colorized the water and make a foam like layers on the surface of water^9^. These layers halted the penetration of sunlight and oxygen into water which finally led to the death of aquatic flora and fauna^10^. Therefore, it is very important to purify these effluent before discharging into the water resources.

Dyes removal from water is one of main environmental problem due to its carcinogenic and mutagenic nature to aquatic life^11^,^12^,^13^. Previous methods such as physical, chemical and biochemical are not useful for the removal of dyes from the effluent^6^. Several methods have been reported in the literature for the de-coloration techniques such as activated carbon^7^,^14^,^15^,^16^,^17^,^18^ however, the advanced oxidation processes (AOPs) effectively used for the degradation and mineralization of dyes into CO_2_ and some inorganic ions. During the removal of dyes the AOPs generated highly reactive species known as ROS (reactive oxygen species i.e. O^•2−^, ^•^OH, or HO^•2^), which possibly invade almost all contaminants. In all known AOPs, the heterogeneous photocatalytic process is the most effective method because of its high availability, low toxicity, inexpensive and diverse nature that might attacked and mineralize a large number of contaminants^13^.

A class of new materials with hydrotalcite like structure known as layered double hydroxide recently attract researchers attention due to their varied application and high anion exchange capacity which make them suitable candidates for catalysis^19^. Hydrotalcite is comparable to brucite like structure Mg(OH)_2_, in which divalent Mg^+2^ is octahedraly sandwich between hydroxyl groups^6^,^20^. LDH has positively charge cations on the surface intercalated negatively charge anion and water molecules^21^. The general formula for LDH is [M^II^_1−x_ M^III^_x_(OH)_2_]^z+^(A^n−^)_z/n_·yH_2_O M^II^ and M^III^ are di- and trivalent cation, (Zn^+2^, Cu^+2^, Al^+3^ and Fe^+3^ etc.) while A^n−^ is the counter anion for cation present in the interlayer of brucite like sheets while x is the (M^II^/M^II^ + M^III^) ratio.

A number of authors have been reported various metal-metal combinations for the synthesis of layered double hydroxide through various techniques for various application. For instance, Gaini *et al.* reported Mg-Al-CO_3_^−2^ for the de-coloration of indigo carmine^6^. Panda *et al.* reported Mg/Al-LDH and study its various factor on its growth such as various metal cation concentration, pH and aging time^22^. Similarly, Shao *et al.* investigated ZnTi-LDH by co-precipitation methods for the de-coloration of methylene blue dyes under the influence of visible light irradiation^23^. The ZnAl-LDH was synthesized by Beak *et al.* through hydrothermal methods^22^. Beside metal-metal combination the LDH was supported on various support to increase its catalytic performance. For instance, carbon nanotubes was used by Li *et al.* as a support for NiAl-LDH through solution method^24^. Similarly, reduced graphene oxide were used as a support for CoAl-LDH for the asymmetric electrochemical capacitors^25^. An efficient MgAl-LDH grown on multi-walled carbon nano tubes MWCNT for CO_2_ adsorption^26^. Composite of NiCoAl-LDH coupled with Ni-Co-carbonate hydroxide supported on graphite paper were used for the asymmetric supercapacitors^27^. In the present work we synthesized Cd-Al and Cd-Sb grafted on activated carbon in order to increase the electronic conductivity through co-precipitation method in which Cd-Al/C were grown in layered double hydroxide morphologies. The synthesized materials were employed for the removal of five dyes AO, MG, CV, CR and MO through adsorption and adsorption assisted photo-degradation.

## Experimental

### Chemicals and reagents

Salt of Al and cadmium nitrate and chloride of cadmium and antimony along with other reagents and dyes mentioned in this manuscript were obtained from Sigma-Aldrich, Ireland. Millipore-Q machine was used for double distilled water, present in chemistry department, King Abdulaziz University Saudi Arabia.

### Synthesis of Cd-Al/C-LDH

Salt of Cd and Al nitrate were well mixed in double distilled water and then mixed with activated carbon through co-precipitation method^19^,^28^,^29^. Briefly salt of Al(NO_3_)_3_ and Cd(NO_3_)_2_ were dissolved thoroughly in double distilled water in 1:3 molar ratio. To this reaction mixture, 10 wt% of activated carbon was added and well dispersed by continuous stirring with the help of magnetic stirrer. To this mixture freshly prepared 0.1 M NaOH solution are added and continuously monitored till pH 9. After this the reaction mixture were placed on a hot plate for 6 h at 60 °C with homogenous stirring. After completion of the reaction the surplus solution is removed and the precipitate was washed thrice with C_2_H_5_OH:H_2_O mixture (8:2). The resultant product was dried in an oven for overnight at 50 °C and store in clean tube for further characterization.

### Synthesis of Cd-Sb/C

The Cd-Sb/C was synthesized in the same way as discuss above for Cd-Al/C-LDH by using SbCl_2_ and CdCl_2_.

### Instrumental analysis and Characterization

FTIR (Thermo Scientific) for functional group analysis and powder X-ray diffractometer (PXRD) with a K*α* radiations (*λ* = 0.154 nm) source were used for the purity and crystallinity of the catalyst. Field emission-scanning electron microscope (FESEM), JEOL (JSM-7600F, Japan) for surface morphology and average size of the particles, while, energy dispersive X-rays spectrometry (EDS) of oxford-EDS system was employed for the elemental composition of the catalyst. X-ray photoelectron spectroscopy (XPS) Thermo Scientific K-Alpha KA1066 spectrometer (Germany) in the range of 0 to 1350 eV was investigated for the elemental analysis as well as for the determination of binding energy in the respective catalyst. The photocatalytic reaction was monitored through Evolution 300 UV–visible spectrophotometer (Thermo scientific). The effect of visible and ultraviolet light on adsorption-degradation process of dyes were observed under visible lamp (OSRAM, 400 watt) and ultraviolet lamp (Smiec Shanghai China, 230 V, 11 watt) respectively. The solar light effect was studied under normal day sunlight in open atmosphere and dark effect in complete absence of light. Photoluminescence emission spectra were confirmed at 320 nm excitation wavelength (fluorescence spectrofluorophotometer), RF-5301 PC, Shimadzu, Japan. The UV–vis diffuse reflectance spectroscopy was recorded by PerkinElmer UV–vis diffuse reflectance spectrophotometer.

### Procedure for dye removal

For this study 0.025% mmol effluent solution of acridine orange (AO), methyl orange (MO), congo red (CR), malachite green (MG) and crystal violet (CV) were prepared. The catalytic activity of Cd-Al/C-LDH and Cd-Sb/C were evaluated against the respective dyes under visible, solar, ultraviolet light, and dark condition. The adjusted dose 10 mg of the respective catalyst were added in 100 mL of 0.025 mmol concentration of dye solution and the gradual decrease in concentration of all the dyes were explored through UV-vis spectrophotometer. The % removal efficiency A.E. (%) of each catalyst was evaluated by using the following equation.


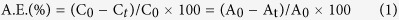


C_0_ represents the original concentration of each dye solution at time = 0, C_t_ is the concentration of dye solution by adding the catalyst after some time = t as indicated in equation (1). Similarly, A_0_ designated the absorbance of the original concentration of the dye solution at time = 0 and A_t_ is the absorbance of dye solution during reaction progress after passing some time = t. Each time 3 mL of the aliquot were taken after specified time and checked the reaction progress by using UV-vis. spectrophotometer.

## Results and Discussion

### Structural characterization of catalyst

**Figure 1:**
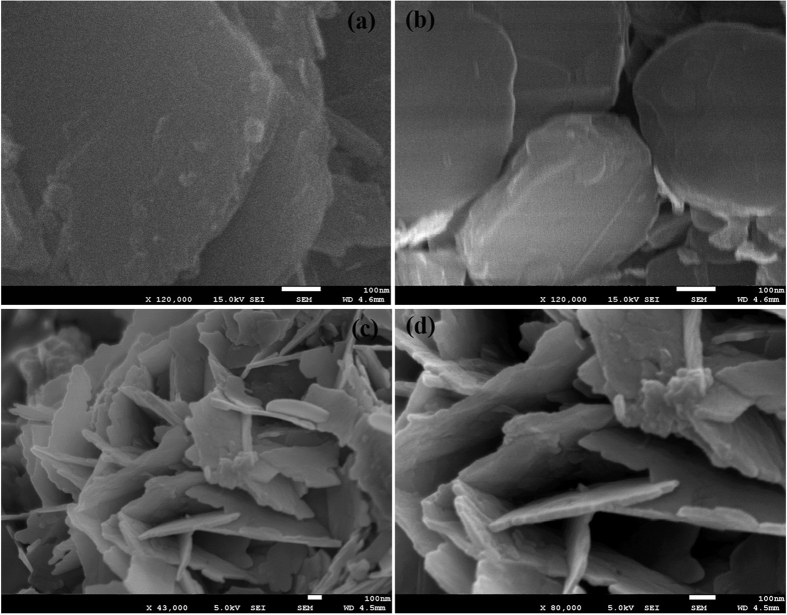
FESEM images of Cd-Al/C-LDH (**a,b**) and Cd-Sb/C (**c,d**) nanosheets.

**Figure 2:**
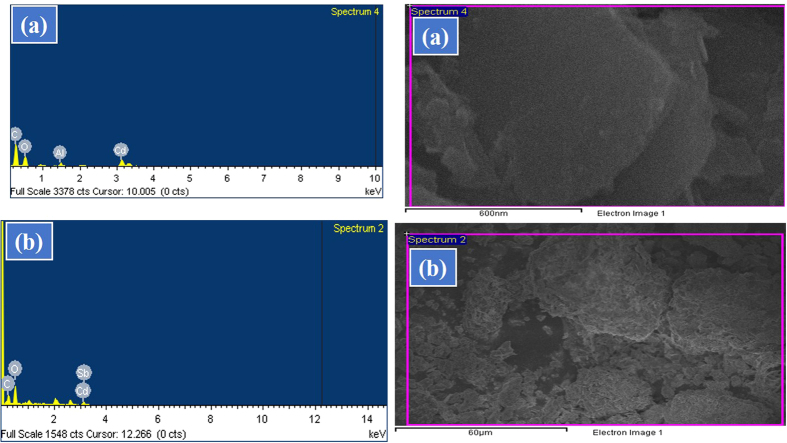
EDS spectrum and plot images of Cd-Al/C-LDH (**a**) and Cd-Sb/C (**b**) nanosheets.

The average size and morphology was scrutinize through FESEM. The FESEM images shows the sheet morphology of Cd-Al/C-LDH (Fig. 1a,b) and Cd-Sb/C (Fig. 1c,d) and the sheets are composed of nanoparticles with average particle size of 50 and 40 nm respectively. In both catalyst small particles are aggregated to form the sheet morphology, however the particle is clearer in Cd-Al/C-LDH than Cd-Sb/C. The elemental analysis was carried out by EDS spectrum which showed the presence of C, O, Al and Cd element in Cd-Al/C-LDH and C, O, Sb and Cd in Cd-Sb/C catalyst (Fig. 2a,b).

**Figure 3:**
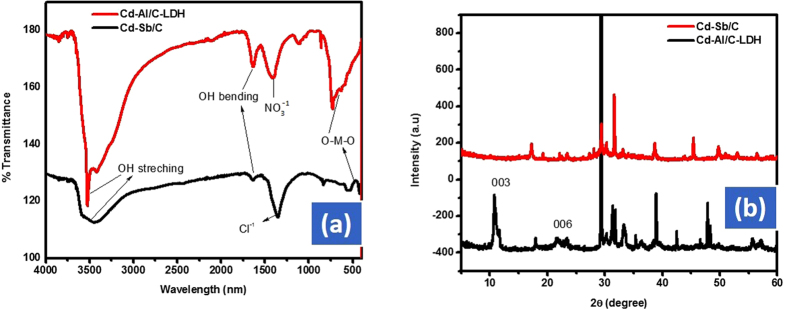
FT-IR spectrum (**a**) and powder XRD spectrum (**b**) of Cd-Al/C-LDH and Cd-Sb/C nanosheets.

The functional groups in the respective catalyst were characterized through FTIR spectroscopy as indicated in Fig. 3a. FTIR analysis exhibited sharp peaks at 460–860 cm^−1^ which were attributed to the M–O, M–O–M and O–M–O bond in the lattice structure of metals of Cd-Al/C-LDH and Cd-Sb/C catalyst^30^. The absorption peaks at 1402 cm^−1^ recommending the presence of NO_3_^−1^ ions in the Cd-Al/C-LDH. The peak1402 at cm^−1^ confirmed that the synthesized catalyst were grown in layered double hydroxide (LDH) morphology. The NO_3_^−1^ ions were present in the interlayer structures of Cd-Al/C-LDH and Cd-Sb/C. Absorption peak at 3520 cm^−1^ exhibited in Cd-Al/C-LDH suggesting the OH stretching vibration in the brucite like layers (LDH). The broadening of this peak suggesting the presence of hydrogen bonding prompted by water molecule present in the interlayer of these hydrocalcite morphology^31^. The OH bending vibration revealed at 1630 cm^−1^ further suggesting the LDH nature of the respective catalyst. It was concluded from the FTIR spectrum that Cd-Al/C-LDH was grown in mixed metal oxide and LDH morphology.

The XRD diffraction pattern shown that Cd-Al/C has grown in mixed oxide and LDH nature while Cd-Sb/C only in the form of oxide. The XRD data suggesting the intercalation of NO_3_^−1^ anions in the brucite like layers of Cd-Al/C-LDH. The characteristic peak for Cd-Al/C-LDH appeared at 2*θ* = 10.8 (003) which was not found in Cd-Sb/C. The 2θ = 23.4 (006) and 2θ = 34.4 (012) suggesting the formation of Cd-Al/C-LDH. The 003 and 006 corresponding to the basal reflection of the successive stacking of brucite like layers^6^. Similarly, the characteristic peaks for Cd-Al/C-LDH appeared C as a doublet at 2θ = 60–62 (110 and 113) further confirm the LDH morphology. The XRD pattern proposes the presence of NO_3_^−1^ anions intercalated in the inner layer of LDH. The remaining XRD peaks correspond to the presence of Cd-Al doped oxide in the Cd-Al/C-LDH. Similarly, the basal peak at 2θ = 31.8 (111), 38.7 (200), 53.1 (220) indicating the formation of CdO while the remaining peaks are due to Sb2O3 in Cd-Sb/C. Figure 3b presented the XRD data of both the catalyst.

**Figure 4:**
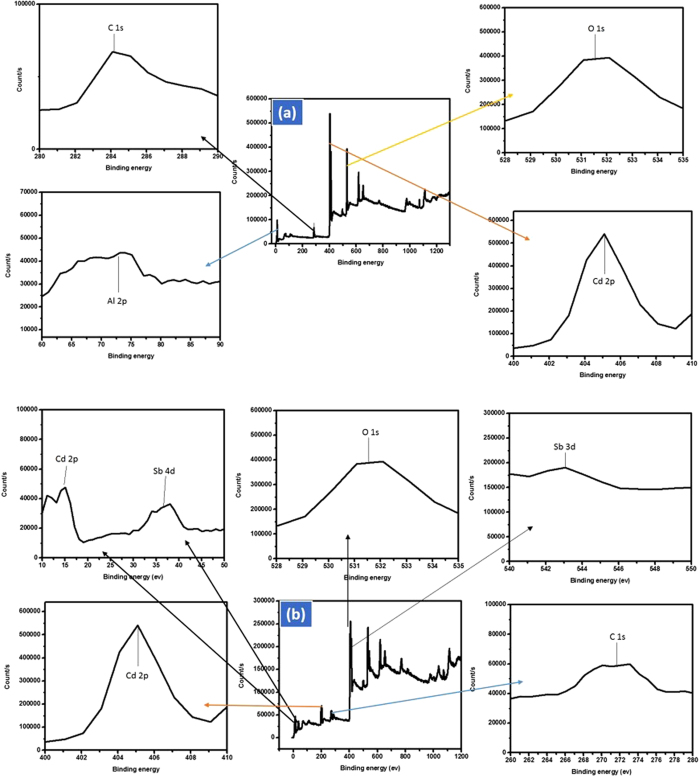
XPS spectrum of Cd-Al/C-LDH (**a**) and Cd-Sb/C (**b**) nanosheets.

The composition analysis and binding energy of Cd-Al/C-LDH and Cd-Sb/C were determined by X-ray photo electron spectroscopy (XPS) from 10–1200 eV as depicts in Fig. 4a,b. The peak at 285 eV suggesting the C1s in Cd-Al/C-LDH. Similarly, Cd2p at 408, Al2p at 75 and O1s were appeared at 530 eV in Cd-Al/C-LDH. The Cd-Sb/C catalyst showed the same pattern for C 1s, O 1s and Cd 2d 275, 530 and 408 eV while the Sb 3d and Sb 4d were exhibited at 620 and 80 eV. This confirm that the respective catalysts are made of O, C, Al and Cd (Cd-Al/C-LDH) and C, Cd, O and Sb in Cd-Sb/C respectively.

**Figure 5:**
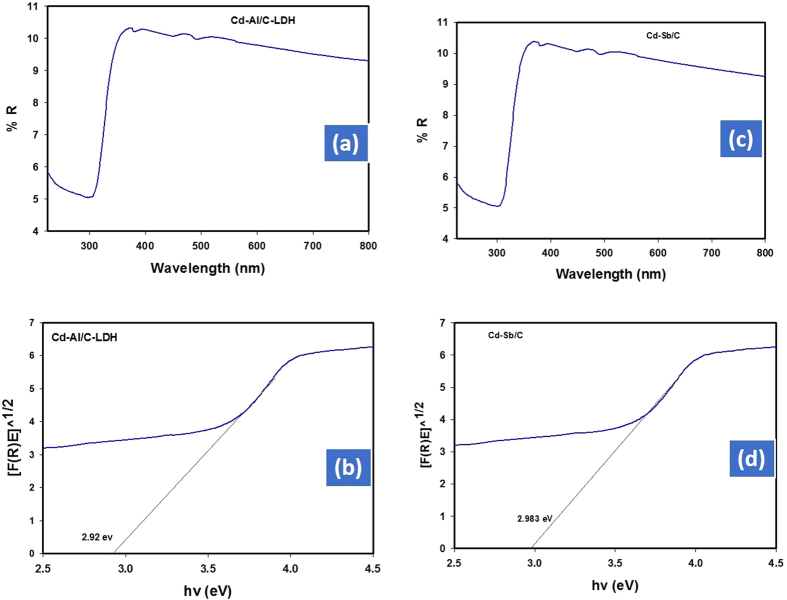
Diffuse reflectance spectra of Cd-Al/C-LDH (**a**), Cd-Sb/C (**c**) and band gap of Cd-Al-/C LDH (**b**), Cd-Sb/C (**d**).

**Figure 6:**
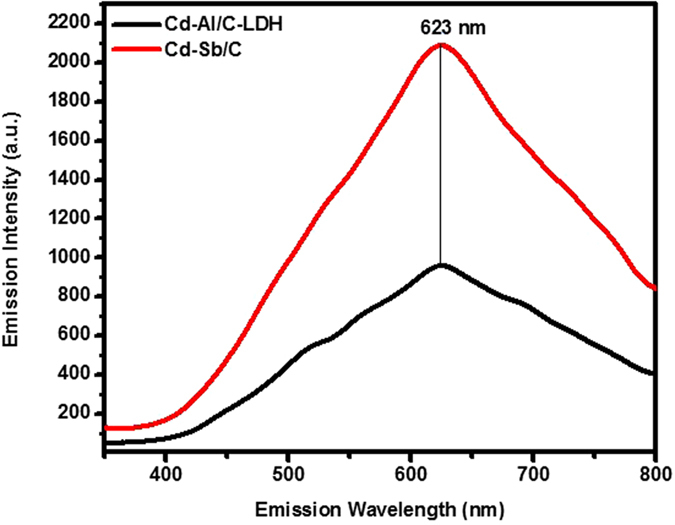
Photoluminescence spectra of Cd-Al/C-LDH and Cd-Sb/C.

The DRS spectra exhibited band gap at 2.92 eV for Cd-Al/C-LDH and 2.983 for Cd-Sb/C. The DRS data indicate that both catalyst work efficiently in the visible range as shown in Fig. 5a,b. The absorption band at 623 nm in photoluminescence spectra of both catalyst further support the efficiency of the respective catalyst in visible range as presented in Fig. 6.

### Photocatalytic activity

The photocatalytic activity of the Cd-Al/C-LDH and Cd-Sb/C was carried out for the de-coloration of five dyes AO, CR, MO, MG and CV under solar, visible, ultraviolet light and dark condition.

### Visible light exposure

Prior to the effect of solar, ultraviolet and dark condition the effect of visible light was study on the de-coloration of cationic and anionic dyes. Both Cd-Al/C-LDH and Cd-Sb/C were evaluated against three different dyes cationic acridine orange (AO) and anionic congo red (CR) and methyl orange (MO) under visible of 400 watt.

### Adjustment of catalytic dose

**Figure 7:**
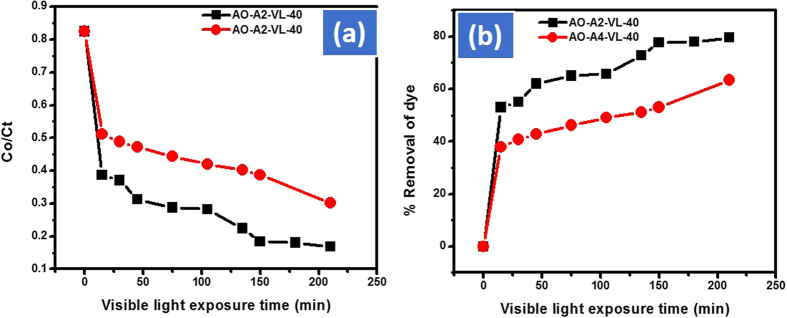
Adsorption assisted photo-degradation of AO in visible light (400 watt) by using 40 mg of A2 and A4 catalyst, concentration decrease of AO (**a**) and % removal of AO (**b**), where AO represent acridine orange, A2, Cd-Al/C-LDH, A4 represent Cd-Sb/C, WOC (without catalyst), and VL (visible light exposure).

**Figure 8:**
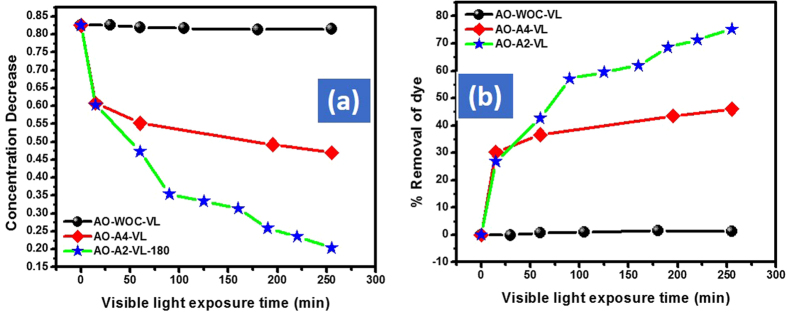
Adsorption assisted photo-degradation of AO in visible light (400 watt) by using 10 mg of A2 and A4 catalyst, concentration decrease of AO (**a**) and % removal of AO (**b**), where AO represent acridine orange, A2, Cd-Al/C-LDH, A4 represent Cd-Sb/C, WOC (without catalyst), and VL (visible light exposure).

**Figure 9:**
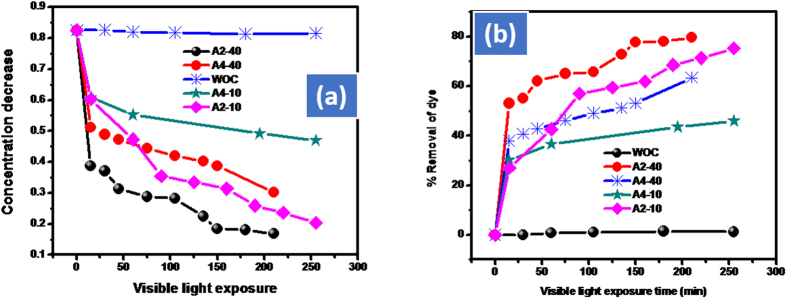
Optimization of catalytic amount in 400 watt visible light irradiation. Where 10 and 40 represent 10 and 40 mg of the catalyst, A2 represent Cd-Al/C-LDH and A4 Cd-Sb/C and WOC without catalyst in graph concentration decrease (**a**) and % removal of acridine orange (**b**).

The catalytic dose was adjusted under visible light by selecting AO dye. Initially, the catalytic doses were adjusted with AO by starting from 100 mg of the respective catalyst in 100 mL of 0.025 mmol of AO. The Cd-Al/C-LDH de-colorize 70% AO while Cd-Sb/C 53% in 1 h. The amount of both the catalyst were decreased to 40 mg in 100 mL of 0.025 mmol of AO in which 63% of AO is removed with Cd-Al/C-LDH and approximately 45% with Cd-Sb/C in 1 h. However, the removal efficiency of dyes is further increased as we increased the contact time. For instance, after 2.5 h under the same condition the % removal of AO with Cd-Al/C-LDH and Cd-Sb/C was approximately 80 and 63% respectively as shown in Fig. 7a,b. Further the amount of the catalysts were decreased to 10 mg in 100 mL of AO. This time the removal efficiency was dropped to 43 and 36% respectively with Cd-Al/C-LDH and Cd-Sb/C in 1 h as shown in Fig. 8a,b. However, by increasing the exposure time of reaction mixture with light the rate of dye removal is also increases and vice versa. During the optimization of the catalyst to dye solution the Cd-Al/C-LDH showed superior activity than Cd-Sb/C (Fig. 9a,b).

**Figure 10:**
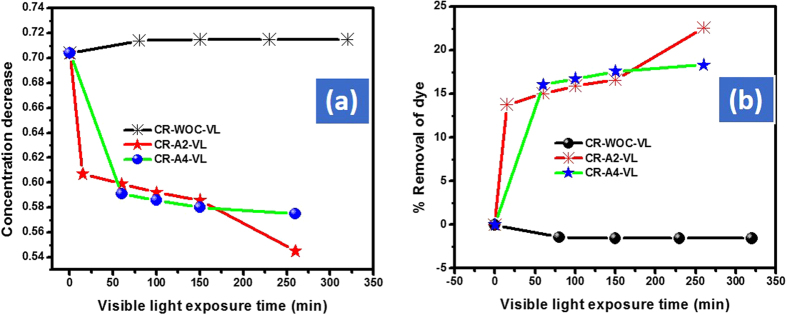
Adsorption assisted photo-degradation of CR in visible light (400 watt) with A2 and A4, concentration decrease of CR (**a**) and % removal of CR (**b**), where CR represent congo red dye, A2, Cd-Al/C-LDH, A4 represent Cd-Sb/C, WOC (without catalyst), and VL (visible light exposure time).

**Figure 11:**
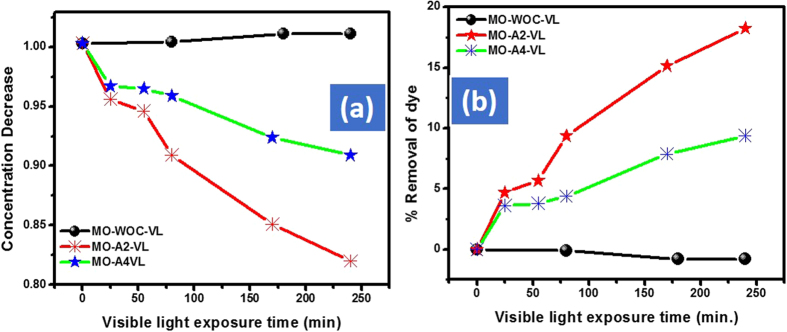
Adsorption assisted photo-degradation of MO in visible light (400 watt) with A2 and A4 concentration decrease of MO (**a**) and % removal of MO (**b**), where MO represent methyl orange dye, A2, Cd-Al/C-LDH, A4 represent Cd-Sb/C, WOC (without catalyst), and VL (visible light exposure time).

Using small amount of the catalyst to dye solution is eco-friendly and therefore, we selected 10 mg of the respective catalyst as an optimized amount for the further detail study. 10 mg of the respective catalyst were further used for the de-colorization of AO, CR and MO. After the first 15 min the % removal efficiency of Cd-Al/C-LDH against AO, CR and MO was 27.0, 13.7 and 4.8% respectively. However, at the same condition the Cd-Sb/C showed 30.7, 3.9 and 3.7% removal efficiency respectively. During the start of the reaction Cd-Sb/C showed slightly good response then Cd-Al/C-LDH. However, onward its activity was much lower than Cd-Al/C-LDH and this might be due to the LDH nature of Cd-Al/C-LDH. By increasing the contact time the % de-coloration of all dyes is increased and it was found that after 200 min, the Cd-Al/C-LDH showed strong response then Cd-Sb/C. For instance, after 200 min the % de-coloration of AO with Cd-Al/C-LDH is 69.4% as compared to Cd-Sb/C which was only 44.0%. It was found that both catalyst showed superior response against cationic dye (AO) as compared to anionic dye (CR and MO) and this is probably due to the large structure of these mentioned anionic dyes. It was also found that for all dyes Cd-Al/C-LDH showed superior performance than Cd-Sb/C. The reaction was also monitored without catalyst with almost no change in dye concentration under visible light, which shows that visible light itself has no role in the de-coloration phenomena. The decrease in the concentration and % removal efficiency of CR and MO are illustrated in Fig. 10a,b (CR), Fig. 11a,b (MO).

### Selectivity of Dye

**Figure 12:**
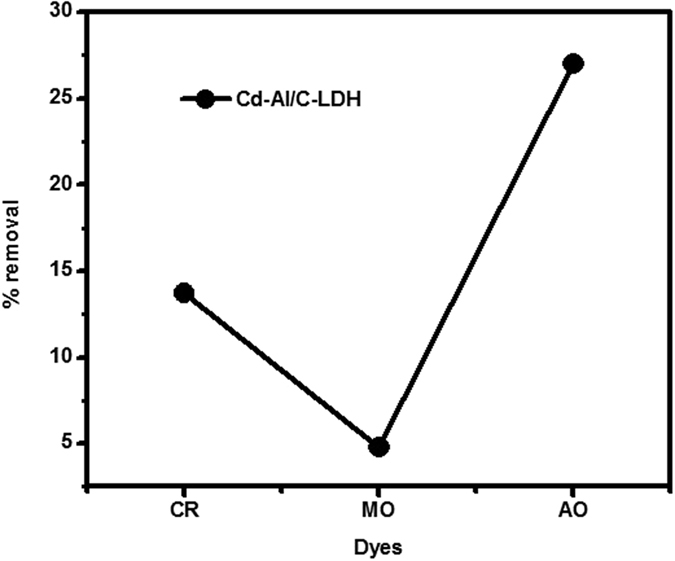
Selective removal of acridine orange (AO) under visible light exposure as compared to congo red (CR) and methyl orange (MO).

Figure 12 showing the selective removal of AO under visible light exposure as compared to CR and MO. Therefore, AO was selected for the further detail study with both catalyst under solar, ultraviolet light and dark condition.

### Sunlight exposure

**Figure 13:**
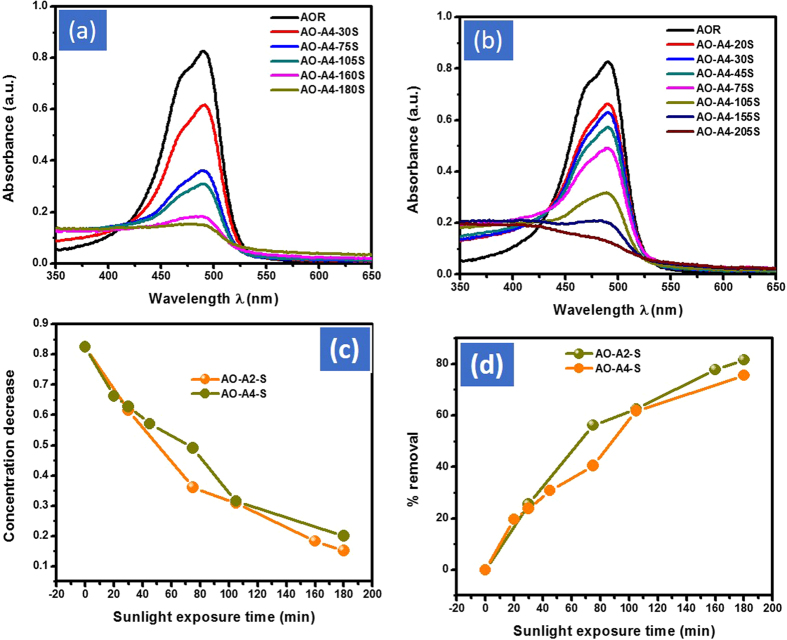
Adsorption assisted photo-degradation of AO in sunlight irradiation. UV-Vis spectrum with A2 (**a**) and A4 (**b**), concentration decrease of AO (**c**) and % removal of AO (**d**), where AO represent acridine orange dye, A2, Cd-Al/C-LDH, A4 Cd-Sb/C, and S represent normal day sunlight exposure time.

Under the same condition both the catalyst were evaluated for the de-coloration of AO in normal day sunlight exposure. The adjusted dose 10 mg of both the catalyst were added in 100 mL of 0.025 mmol of AO solution. The Cd-Al/C-LDH showed faster and better response then Cd-Sb/C in sunlight exposure. After the first 15 min of experiment the Cd-Al/C-LDH showed 13.5 while Cd-Sb/C showed 18.5% de-coloration of AO. Similarly, after 180 min the % de-coloration of AO with Cd-Al/C-LDH increased from 13.5 to 82.2% and Cd-Sb/C from 18.5 to 76.0%. This inferred the better performance of both catalyst with the passage of time as shown in the inset of Fig. 13a–d.

Under visible and solar light irradiation it was concluded that cationic dye AO is selectively removed with the corresponding catalyst. It was also confirmed that cationic dye AO is predominantly removed in solar light as compared to visible light and it could possibly be the combine effect of visible, UV and IR regions in solar light. Therefore, we studied two more cationic dyes malachite green (MG) and crystal violet (CV) under the same condition and with same catalyst in solar light.

**Figure 14:**
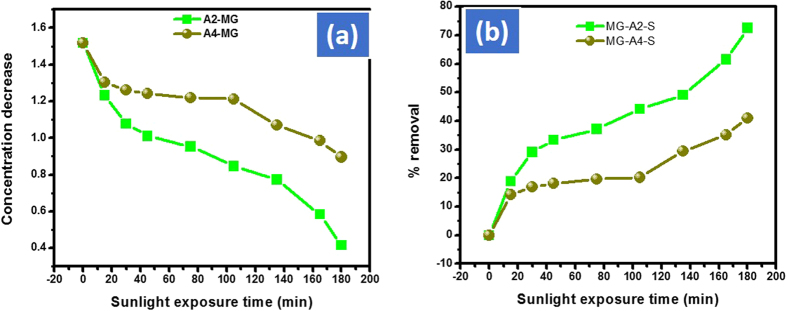
Concentration decrease (**a**) and % removal of MG (**b**) with A2 and A4 catalyst. Where MG represent malachite green dye, A2 Cd-Al/C-LDH and A4 Cd-Sb/C catalyst and S represent normal day sunlight exposure.

**Figure 15:**
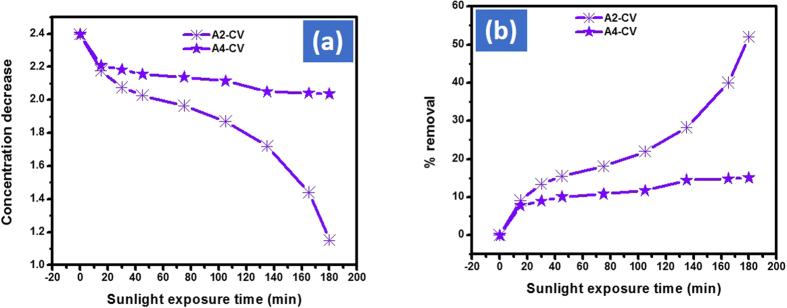
Concentration decrease (**a**) and % removal of CV (**b**) with A2 and A4 catalyst. Where CV represent crystal violet dye, A2 Cd-Al/C-LDH and A4 Cd-Sb/C catalyst and S represent normal day sunlight exposure.

After 180 mins under the same condition Cd-Al/C-LDH showed 82, 72 and 52% removal efficiency against AO, MG and CV dyes respectively. Similarly, Cd-Sb/C indicating approximately 75, 41 and 15% removal efficiency against AO, MG and CV respectively. The decrease in original concentration and percent removal of MG and CV with Cd-Al/C-LDH and Cd-Sb/C are depicted in Figs 14a,b and 15a,b respectively.

**Figure 16:**
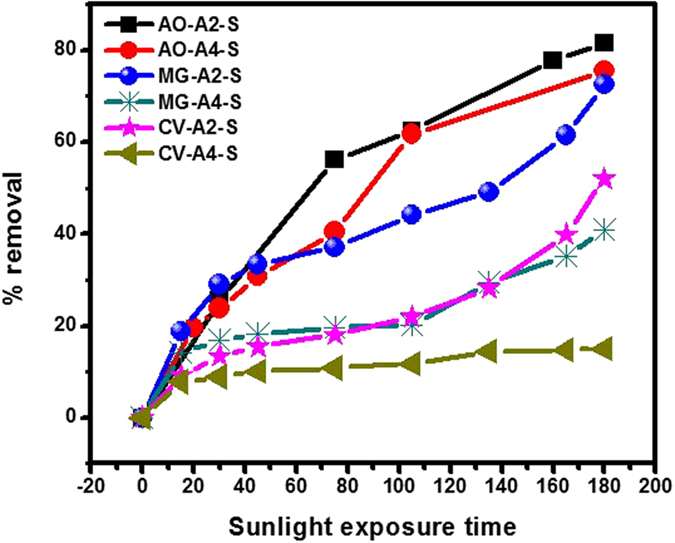
% removal of AO, MG and CV in sunlight irradiation with Cd-Al/C-LDH (A2) and Cd-Sb/C (A4).

The catalytic activity of the respective catalysts were excellent, better and good for AO, MG and CV respectively. Cd-Al/C-LDH showed stronger catalytic activity over Cd-Sb/C catalyst and selectively de-colorized AO over MG and CV as presented in Fig. 16. Therefore, we select AO for further study under ultraviolet light and dark condition.

### Ultraviolet light exposure

**Figure 17:**
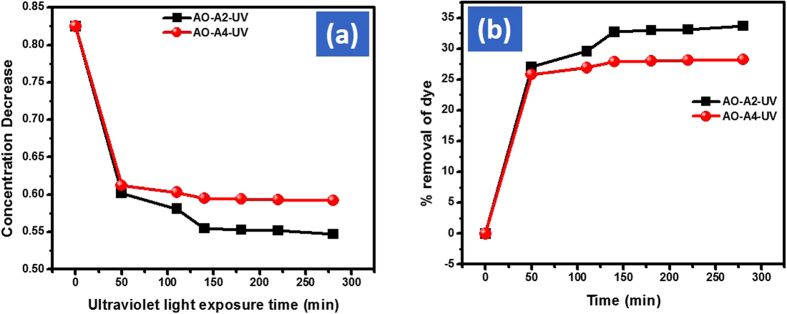
Adsorption of AO in ultraviolet light by A2 and A4, concentration decrease of AO (**a**) and % removal of AO (**b**), where AO represent acridine orange dye, A2, Cd-Al/C-LDH, A4 Cd-Sb/C, and U represent ultraviolet light irradiation.

UV lamp (230 volt, 11 watt) was used for the de-coloration of 0.025 mmol AO solution. Keeping the same condition as adjusted for visible and solar light, 10 mg of both the catalyst were used for the de-coloration of 100 mL of 0.025 mmol AO solution. After the first 15 min of exposure time the % removal efficiency of Cd-Al/C-LDH and Cd-Sb/C against AO was 8.5 and 7.5% respectively. However, after 200 min, the removal efficiency of AO (%) was increased to 36.3% with Cd-Al/C-LDH and 29.1% with Cd-Sb/C. Like visible and solar light the Cd-Al/C-LDH showing superior removal for AO as compared to Cd-Sb/C. The decrease in concentration and % removal efficiency of AO under ultraviolet light exposure are presented in the inset of Fig. 17a,b.

### Dark condition exposure

**Figure 18:**
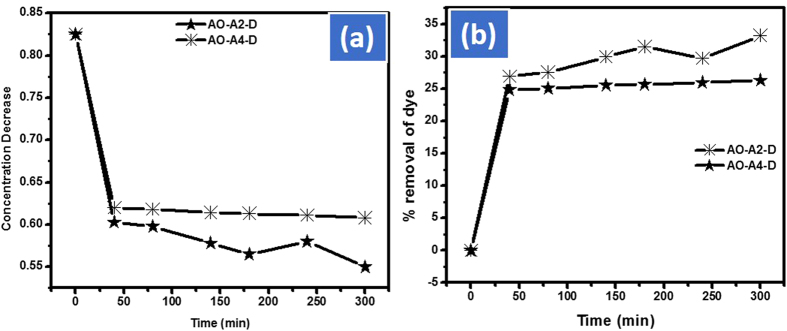
Adsorption of AO in dark condition by A2 and A4, concentration decrease of AO (**a**) and % removal of AO (**b**), where AO represent acridine orange dye, A2, Cd-Al/C-LDH, A4 Cd-Sb/C, and D represent complete dark condition.

Prior to solar and ultraviolet light the effect of dark was studied for both catalyst to know the adsorption or degradation phenomena. The adjusted dose 10 mg of the respective catalyst were put in a beaker containing 100 mL of 0.025 mmol AO solution by providing complete dark condition. After the first 15 min of the reaction progress the Cd-Al/C-LDH displayed 11.0% and Cd-Sb/C 10.9% removal efficiency. However, after 200 min, the Cd-Al/C-LDH was showing 31.0% and Cd-Sb/C 26.0% removal of AO as indicated in Fig. 18a,b.

After the detailed study for the de-coloration of dyes (AO, CR, MO, MG, CV) under solar, visible, ultraviolet light and absence of light, it was concluded that cationic dyes removed preferentially then anionic dyes. Among the cationic dyes (AO, MG and CV) AO was predominantly removed by both catalyst in solar light. However, the removal efficiency of MG and CV are also good. It was inferred that AO adsorbed in the absences of light and adsorbed plus degraded in ultraviolet, visible and solar light. The adsorption process is triggered by the presence of activated carbon in the respective catalyst. The dyes is adsorbed on the catalyst and then degraded as the reaction proceeded.

#### Kinetic study of the reaction

**Figure 19:**
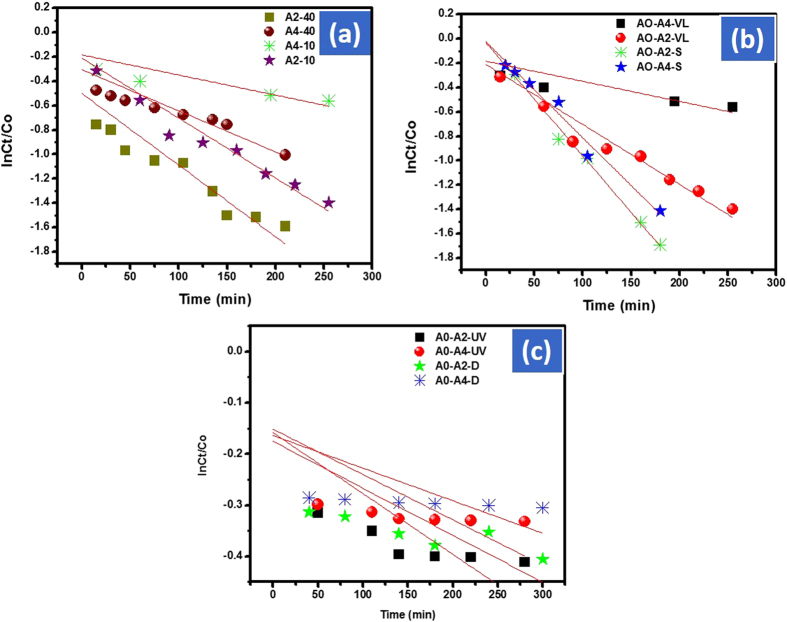
Langmuir adsorption isotherm of AO with catalyst A2 and A4 in solar (S) and visible light (VL) where AO represent acridine orange, A2, Cd-Al/C-LDH and A4 Cd-Sb/C.

The kinetics of the reaction was determined by applying pseudo first order kinetics (lnC_t_/C_o_). This model was applied to compare the rate of Cd-Al/C-LDH and Cd-Sb/C in visible light by using different amount of the catalyst against the removal of AO. This equation also applied to investigate the rate of reaction in different parameters like dark, ultraviolet, visible and solar light. The rate of the reaction was determined by plotting lnC_t_/C_o_ should be subscript verses time. The model showed the highest rate of Cd-Al/C-LDH then Cd-Sb/C in all conditions. The rate of reaction is directly related to the amount of catalyst. For instance, at 40 mg of Cd-Al/C-LDH the rate of reaction was 5.88 × 10^−3^ mol L^−1^ min^−1^ as compared to 4.92 × 10^−3^ mol L^−1^ min^−1^ obtained when 10 mg of the same catalyst was used under solar light. The same trends was observed in visible light. However, the rate of degradation is slow from solar light as inspected in Fig. 19a. In all other parameters Cd-Al/C-LDH showed higher rate of reaction then Cd-Sb/C as indicated in the inset of Fig. 19b,c.

#### Catalytic recyclability

**Figure 20:**
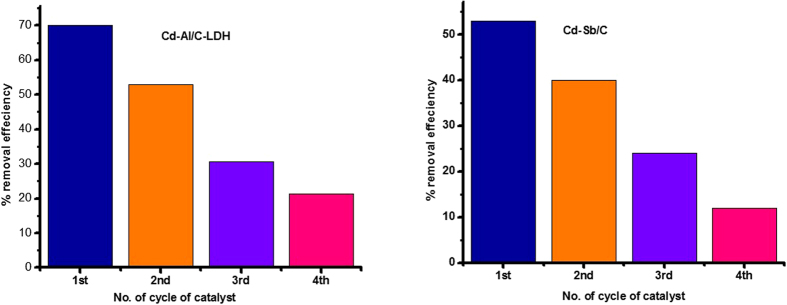
Catalytic activity of Cd-Al/C-LDH and Cd-Sb/C catalyst against AO under visible light exposure of 400 watt. Each cycle lasted for 1 h.

**Figure 21:**
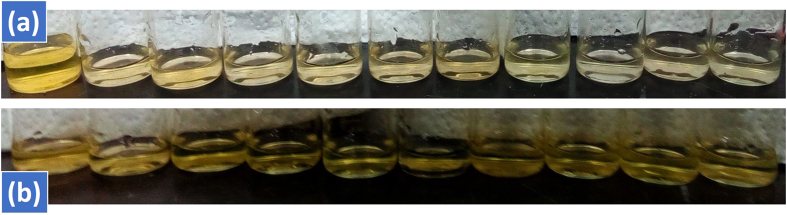
Gradual decrease in AO color with Cd-Al/C-LDH (**a**) and Cd-Sb/C (**b**) under visible light irradiation.

The catalytic recyclability is critical issue while carrying catalysis. Most of the catalyst become de-activated after first or second cycle. During the recyclability of the catalyst 100 mg of the respective catalyst was added in 100 mL (0.025 mmol) of AO under visible light of 400 watt. After one hour, the reaction was stopped and 3 mL of the aliquot was taken through a clean syringe and examined in UV-vis. spectrophotometer. After this the catalyst was separated through filtration process and the filtrate was washed thrice with acetone. The recovered washed catalyst (obtained from first run) was used in the next cycle for 100 mL of 0.025 mmol AO solution without drying or heating. The reaction mixture was again placed for 1 h under visible light exposure. Similarly, after one hour the reaction was stopped for monitoring the decrease in concentration and catalytic activity through UV-vis spectrophotometer. The catalyst is likewise separated carefully and washed thrice with acetone and used in the third cycle for 100 mL of 0.025 mmol AO. The same practice recurred four times for Cd-Al/C-LDH and Cd-Sb/C. In each cycle the reaction mixture was placed under visible light for one hour. The Cd-Al/C-LDH indicated 70% removal efficiency in the first cycle and decreases to 53, 30 and 21% in second, third and four cycle respectively. Similarly, Cd-Sb/C indicated 52% AO removal in the first cycle which was decreased to 40, 25 and 11% in second, third and fourth cycle respectively. It was also found that Cd-Al/C-LDH was remained active. However, Cd-Sb/C lost its activity after forth cycle. Both the catalyst showed good recyclability and durability as shown in the inset of Fig. 20a,b. The gradual de-coloration of AO dye with the respective catalyst are indicated in Fig. 21.

#### Structural feature of dyes and photocatalytic activity

**Figure 22:**
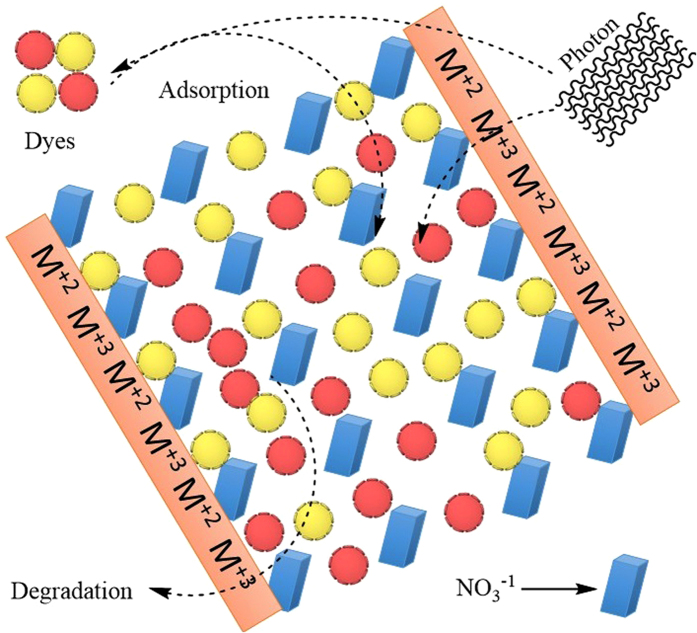
Schematic view of adsorption assisted photo-degradation of dyes.

The structural features of dyes play a significant role in dyes light. However, it is necessary to select specific materials according to the functional groups in dyes. Dyes degradation generally undergoes through breakage of various functional group i.e. cleavage of the aromatic, C–S bond breaking occurred between an aromatic ring and the sulphur of a sulphonyl group. Similarly, other functional group such as C–C, C–N and azo bond breakage will also happened during dyes degradation^31^. Both catalyst showed better performance for cationic dyes AO, MG and CV then anionic dyes CR and MO. Among the cationic dyes AO selectively removed as compared to MG and CV. The higher adsorption assisted photo-degradation of AO was probably due to the cleavage of C–C and C–N bond of aromatic ring and their non-bulky nature. While, lesser removal of CR and MO were due to the presence of azo group and large bulky groups which interfere with the charge transfer during de-coloration process. The LDH has layered double structure with upper cationic and inner anionic layers. The schematic phenomena of adsorption assisted photo-degradation of dyes is shown in Fig. 22.

## Conclusion

The inexpensive and diverse morphology make layered double hydroxide a suitable candidate in the field of catalysis. Cd-Al/C-LDH and Cd-Sb/C were synthesized through co-precipitation method. Both the catalyst showed a narrow band gap which indicated its effectiveness in visible region. The PL also showed a peak at 623 nm for both catalyst which showed its efficiency in visible range. Both catalyst grown in nanosheet morphologies. The Cd-Al/C has LDH nature as confirmed from FTIR and XRD. The respective catalyst were used for the de-coloration and mineralization of organic dyes from the effluent under solar, ultraviolet, visible light and dark condition. The Cd-Al/C-LDH shown better catalytic activity in all conditions and this is probably due to its layered double hydroxide nature. The rate of reaction was determined from Langmuir isotherm indicating high rate of Cd-Al/C-LDH as compared to Cd-Sb/C. A predominate degradation and negligible adsorption was found in solar light and an equal percentage of degradation and adsorption were found in visible light. Similarly, adsorption was observed in dark condition and ultraviolet light. The Cd-Al/C-LDH and Cd-Sb/C showed good recyclability, durability and easy separation. These results showed the high activity and the ease in separation of the catalyst which are routinely encounter in nanocatalysis.

## Additional Information

**How to cite this article**: Khan, S. A. *et al.* Layered double hydroxide of Cd-Al/C for the Mineralization and De-coloration of Dyes in Solar and Visible Light Exposure. *Sci. Rep.* **6**, 35107; doi: 10.1038/srep35107 (2016).

**Publisher’s note:** Springer Nature remains neutral with regard to jurisdictional claims in published maps and institutional affiliations.
